# R- Is Superior to S-Form of α-Lipoic Acid in Anti-Inflammatory and Antioxidant Effects in Laying Hens

**DOI:** 10.3390/antiox11081530

**Published:** 2022-08-05

**Authors:** Qingxiu Liu, Wenxiang Li, Shimeng Huang, Lihong Zhao, Jianyun Zhang, Cheng Ji, Qiugang Ma

**Affiliations:** State Key Laboratory of Animal Nutrition, College of Animal Science and Technology, China Agricultural University, Beijing 100193, China

**Keywords:** S-form of α-lipoic acid, R-form of α-lipoic acid, oxidized fish oil, antioxidant, laying hen

## Abstract

The development of single enantiomers with high efficiency and low toxic activity has become a hot spot for the development and application of drugs and active additives. The aim of the present study was to investigate the effectiveness of the application of α-lipoic acid with a different optical rotation to alleviate the inflammation response and oxidative stress induced by oxidized fish oil in laying hens. Sixty-four 124-week-old Peking Red laying hens were randomly allocated to four groups with eight replicates of two birds each. The normal group was fed basal diets supplemented with 1% fresh fish oil (FO), and the oxidative stress model group was constructed with diets supplemented with 1% oxidized fish oil (OFO). The two treatment groups were the S-form of the α-lipoic acid model with 1% oxidized fish oil (OFO + S-LA) and the R-form of the α-lipoic acid model with 1% oxidized fish oil (OFO + R-LA) added at 100 mg/kg, respectively. Herein, these results were evaluated by the breeding performance, immunoglobulin, immune response, estrogen secretion, antioxidant factors of the serum and oviduct, and pathological observation of the uterus part of the oviduct. From the results, diets supplemented with oxidized fish oil can be relatively successful in constructing a model of inflammation and oxidative stress. The OFO group significantly increased the levels of the serum inflammatory factor (TNF-α, IL-1β, IL-6, and IFN-γ) and the oxidative factor MDA and decreased the activity of the antioxidant enzyme (T-AOC, T-SOD, GSH-Px, GSH, and CAT) in the oviduct. The addition of both S-LA and R-LA significantly reduced the levels of serum inflammatory factors (TNF-α, IL-1β, IL-6, and IFN-γ), increased the activity of antioxidant indexes (T-AOC, T-SOD, GSH-Px, GSH, and CAT), and decreased the MDA contents in the serum and oviduct. Meanwhile, the supplementation of S-LA and R-LA also mitigated the negative effects of the OFO on the immunoglobulins (IgA and IgM) and serum hormone levels (P and E2). In addition, it was worth noting that the R-LA was significantly more effective than the S-LA in some inflammatory (IL-1β) and antioxidant indices (T-SOD, GSH, and CAT). Above all, both S-LA and R-LA can alleviate the inflammation and oxidative damage caused by oxidative stress in aged laying hens, and R-LA is more effective than S-LA. Thus, these findings will provide basic data for the potential development of α-lipoic acid as a chiral dietary additive for laying hens.

## 1. Introduction

Egg production is one of the main factors limiting the longevity and economic efficiency of laying hens [1]. Egg production in laying hens is mainly associated with ovarian and oviductal health, accompanied by endocrine hormonal changes [2]. However, in the current commercial farming model, various stressors and continuous high production can cause oxidative damage to the oviduct of laying hens [3]. Over time, oxidative damage to the oviduct accumulates in the organism and adversely affects the production performance and immune system, ultimately posing a serious threat to the reproductive health of laying hens [4].

Fish oil is an oleaginous substance rich in a variety of n-3 polyunsaturated fatty acids, but it is highly susceptible to oxidation, generating lipid oxidation products [5]. Currently, the harmful effects of oxidized fish oil on breeding production have been widely reported [6,7]. In several experiments in poultry, mice, and piglets, the dietary addition of oxidized fish oil was found to exacerbate oxidative stress and damage in the organism by reducing antioxidant-related enzymes [8,9,10]. Therefore, oxidative stress models constructed by the dietary addition of oxidized fish oil have been used extensively in animal experiments.

Alpha-Lipoic acid (ALA) has strong natural antioxidant properties and has been widely used as medicine or health-care food [11,12]. Natural α-lipoic acid is found in many foods, such as spinach, cauliflower, potatoes, animal meat, liver, heart, and kidneys. Among the research on human diseases, ALA plays an important role in the mitochondrial bioenergetic response and can be used in managing liver disease, diabetes and its complications, AIDS, heart disease, retinopathy, neuropathy, and other vascular diseases [13,14]. In the study of poultry health, ALA increased the anti-inflammation and antioxidant capacity in serum and liver, as well as reduced the level of lipid peroxidation in vivo under normal dietary conditions in broilers [15].

**Figure 1:**
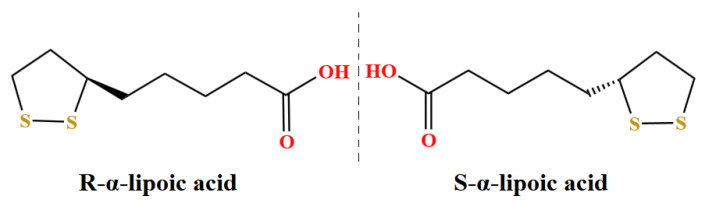
Chemical structure of S-form of α-lipoic acid and R-form of α-lipoic acid.

ALA is the chiral molecule of a drug having an asymmetric carbon atom existing in both R- and S-forms (Figure 1) [16,17]. A study has shown the approximately twofold higher bioavailability of the R-form of α-lipoic acid (R-LA), as compared to the S-form of α-lipoic acid (S-LA) [18]. In a dose/response curve model of R-LA and S-LA in the working rat heart during reoxygenation, the R-enantiomer was found to be more superior in the enhancement of the aortic flow [19]. Moreover, in a model of sciatic nerve injury in hypertensive rats, R-LA was more active than S-LA in terms of analgesic and neuroprotective efficacy [20,21]. This may be related to the better recognized dextrorotatory enantiomer by enzymes, as it was found that the maximum plasma concentration (Cmax) is approximately 40–50% higher with R-LA than with S-LA at the same dose [11]. However, there are still gaps in the research on the effects of different enantiomers of ALA to alleviate the anti-inflammatory and antioxidant properties of the organism and oviduct in aged laying hens.

Therefore, in this research, we first constructed an oxidative stress model using oxidized fish oil on aged laying hens, then investigated the antioxidant and anti-inflammatory capacity of different enantiomers of ALA by observing the effect of R-LA and S-LA in alleviating the oxidative stress and inflammatory response in the oviduct.

## 2. Materials and Methods

The handling protocol of animals in this study was approved by the Institutional Animal Care and Use Committee of the China Agricultural University (Permit number: Aw71202202-1-1).

### 2.1. Materials

Fresh fish oil (FO) without any antioxidant was purchased from Baiyang Investment Group, Inc (Nanning, China) and stored in a refrigerator at −20 °C prior to their addition to feed. Oxidized fish oil preparation process: add 30 mg/kg Fe^2+^, 15 mg/kg Cu^2+^, 600 mg/kg H_2_O_2_, and 0.3% water for fresh fish oil. After thorough mixing, the oxidized fish oil with a peroxide value (POV) of 1200.10 meq O_2_/kg was obtained by stirring at 37° for 168 h with oxygen. S-form of α-lipoic acid (S-LA, >97% (HPLC), CAS RN: 1022-27-6) was purchased from aladdin (Shanghai, China) and R-form of α-lipoic acid (R-LA, >98.0% (T) (HPLC), CAS RN: 1200-22-2) was purchased from Tokyo Chemical Industry (Tokyo, Japan).

### 2.2. Experimental Design

A total of 64 124-week-old Peking Red laying hens (Yukou Poultry Co., Ltd. of Beijing, Beijing, China) were randomly divided into four treatments: the basal diet supplemented with 1% fresh fish oil group (CON), the basal diet supplemented with 1% oxidized fish oil group (OFO), the basal diet supplemented with 1% oxidized fish oil plus 100 mg/kg S-LA group (OFO + S-LA), and the basal diet supplemented with 1% oxidized fish oil plus 100 mg/kg R-LA group (OFO + R-LA), with eight replicates of two hens each. During the experiment period, the hens were fed according to the basal corn-soybean meal diet (NYT33-2004) (Supplementary Table S1).

### 2.3. Sample Collection

During the four weeks of the experiment, the breeding performance such as weekly egg laying rate, egg weight, egg production, feed intake, weight of laying hens, and feed-to-egg ratio were recorded and calculated. At the end of 127 weeks of age, one laying hen was randomly selected for each replicate. Wing venous blood was collected, and serum was obtained by centrifugation of a respective blood sample at 3000 r/min for 15 min at 4 °C and stored at −80 °C until further analysis. The hens were sacrificed after collection of blood samples. The uterus part of oviduct (the more expanded part of the oviduct, about 10–20 cm from the cloaca) was isolated, washed with PBS, and separated into two parts. One part was weighted and diluted with nine-times volumes of sterile ice-cold normal saline (0.9%) and then homogenized using a hand-held glass homogenizer. The tissue supernatants were collected by centrifuging at 3500× *g* for 10 min at 4 °C, and the concentration of protein was determined by a BCA protein assay kit according to the manufacturer’s instruction (Pierce, Rockford, IL, USA) and stored at −80 °C for further study [22].

### 2.4. Indicator Test

The immune and inflammatory states are determined by the plasma immunoglobulin G (IgG), immunoglobulin A (IgA), immunoglobulin M (IgM), tumor necrosis factor α (TNF-α), interleukin 1β (IL-1β), interleukin 6 (IL-6), and interferon γ (IFN-γ). The quantifications of immune factors in plasma samples were performed using commercial kits (Nanjing Jiancheng Bioengineering Institute, Nanjing, China).

Serum hormone levels are indicated by estradiol (E2) and progesterone (P). The quantifications of hormone levels in serum were measured with commercial kits (Nanjing Jiancheng Bioengineering Institute, Nanjing, China).

The redox status was evaluated by measurement of the total antioxidant capacity (T-AOC), total superoxide dismutase (T-SOD), glutathione peroxidase (GSH-Px), glutathione (GSH), catalase (CAT), and malondialdehyde (MDA) in serum and uterus part. The quantifications of antioxidant factors in samples were performed using commercial kits (Nanjing Jiancheng Bioengineering Institute, Nanjing, China). The assays were carried out according to the manufacturer’s instructions, and the absorbance levels were read at 540 and 405 nm, respectively, with a microplate reader (Nanjing Jiancheng Bioengineering Institute, Nanjing, China).

### 2.5. Hematoxylin-Eosin Staining and Histopathology Analyses

Another uterine portion of the oviduct was evaluated for histopathological damage. The sections were first fixed in 4% paraformaldehyde, then embedded in paraffin, cut into 5 μm micro-sections, mounted on glass slides, and finally stained with eosin Y solution (Solarbio, Beijing, China) and hematoxylin (Solarbio, Beijing, China). Hematoxylin and eosin (H&E)-stained paraffin sections were observed under bright field on a Zeiss Axio Imager microscope as outlined previously. Microscopic oviduct damage was observed in images using the measurement tool on Case Viewer software at 100× magnification.

### 2.6. Statistical Analysis

All data were statistically analyzed using SPSS 25.0 (SPSS Science, Chicago, IL, USA). Comparisons of means among groups were conducted by one-way ANOVA, followed by Tukey’s post hoc test. Differences were regarded as significant at *p* < 0.05 and highly significant at *p* < 0.01.

## 3. Results

### 3.1. Effect of S-LA and R-LA on Reproductive Performance of Laying Hens under Oxidative Stress

As shown in Table 1, compared with the control group, there were no significant differences in all the breeding production factors (*p* > 0.05). Compared with the OFO group, only the egg laying rate in the OFO + R-LA group was significantly increased (*p* < 0.05), but there were no significant differences in the egg weight, egg production, feed intake, weight of laying hens, and feed/egg radio (*p* > 0.05).

### 3.2. Effect of S-LA and R-LA on Immunoglobulin of Laying Hens under Oxidative Stress

**Figure 2:**
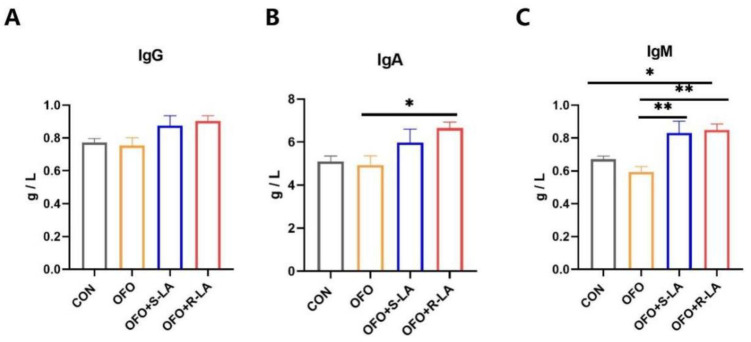
Effect of different treatments on immunoglobulin of laying hens. (**A**) Immunoglobulin G (IgG); (**B**) immunoglobulin A (IgA); (**C**) immunoglobulin M (IgM). * Indicates minimum significant differences (*p* < 0.05), and ** indicates highly significant differences (*p* < 0.01).

The serum immunoglobulin levels after the different treatments are shown in Figure 2. Compared with the CON group, the serum immunoglobulin levels in the OFO group showed no significant change (*p* > 0.05). Compared with the OFO group, the dietary addition of the S-LA significantly increased the IgA levels (*p* < 0.05), and the dietary addition of the R-LA significantly increased the levels of IgA and IgM (*p* < 0.01). In addition, the IgM levels in the OFO + R-LA group were higher than that in the CON group (*p* < 0.05).

### 3.3. Effect of S-LA and R-LA on Serum Inflammatory Factors of Laying Hens under Oxidative Stress

**Figure 3:**
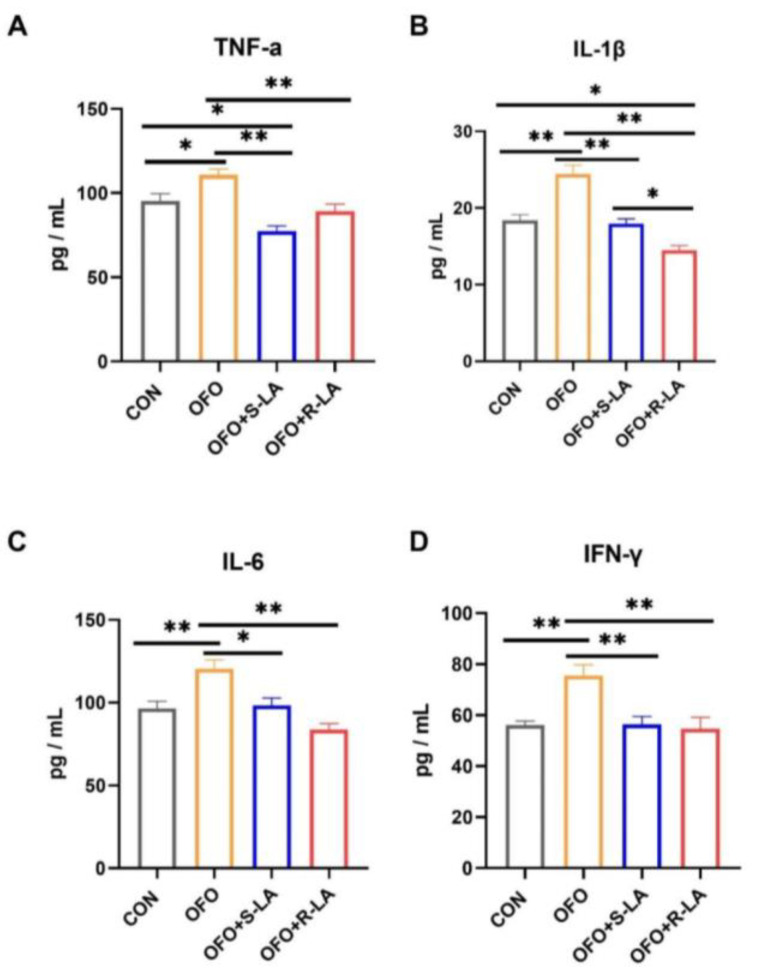
Effect of different treatments on serum inflammatory factors of laying hens. (**A**) Tumor necrosis factor α (TNF-α); (**B**) interleukin 1β (IL-1β); (**C**) interleukin 6 (IL-6); (**D**) interferon γ (IFN-γ). * Indicates minimum significant differences (*p* < 0.05), and ** indicates highly significant differences (*p* < 0.01).

To evaluate the effects on the immune responses, the serum levels of the TNF-α, IL-1β, IL-6, and IFN-γ were assayed by ELISA. Figure 3 shows that, compared to the CON group, the serum levels of the TNF-α, IL-1β, IL-6, and IFN-γ in the OFO group were significantly increased (*p* < 0.01). Compared to the OFO group, the serum TNF-α, IL-1β, IL-6, and IFN-γ levels in the OFO + S-LA and OFO + R-LA group were significantly suppressed (*p* < 0.01). In addition, the TNF-α in the OFO + S-LA group and the IL-1β in the OFO + R-LA group were significantly decreased compared to the CON group (*p* < 0.05). Compared with the OFO + S-LA group, the IL-1β level in the OFO + R-LA group was significantly decreased (*p* < 0.05).

### 3.4. Effect of S-LA and R-LA on Serum Estrogen of Laying Hens under Oxidative Stress

**Figure 4:**
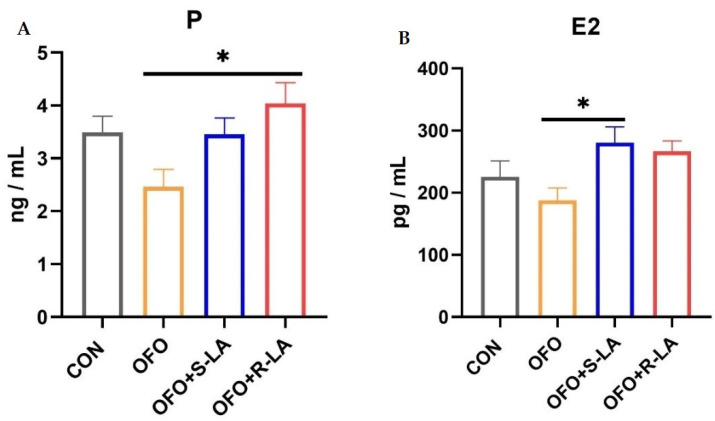
Effect of different treatments on serum estrogen of laying hens. (**A**) Progesterone (P); (**B**) estradiol (E2). * Indicates minimum significant differences (*p* < 0.05).

As shown in Figure 4, the serum estrogen content was not significantly different between the OFO group and the OFO group (*p* > 0.05). However, compared with the OFO group, the P content in the OFO + R-LA group and the E2 content in the OFO + S-LA group were all significantly increased (*p* < 0.05).

### 3.5. Effect of S-LA and R-LA on Serum Antioxidant of Laying Hens under Oxidative Stress

**Figure 5:**
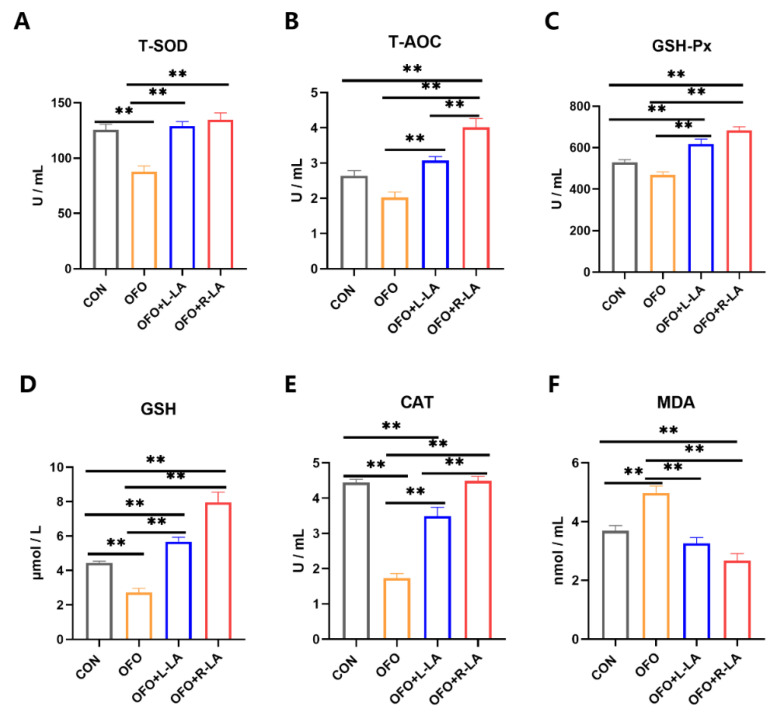
Effect of different treatments on serum antioxidant of laying hens. (**A**) Total superoxide dismutase (T-SOD); (**B**) total antioxidant capacity (T-AOC); (**C**) glutathione peroxidase (GSH-Px); (**D**) glutathione (GSH); (**E**) catalase (CAT); (**F**) malondialdehyde (MDA). ** indicates highly significant differences (*p* < 0.01).

The addition of the oxidized fish oil significantly increased the serum T-AOC, T-SOD, GSH-Px, GSH, and CAT activities and decreased the MDA levels compared to the addition of fresh fish oil in Figure 5 (*p* < 0.01). Compared with the OFO group, the activities of the serum T-AOC, T-SOD, GSH-Px, GSH, and CAT were all significantly increased, and the serum MDA level was significantly decreased in the OFO + S-LA group and the OFO + R-LA group (*p* < 0.01). Moreover, compared with the CON group, the CAT activities in the OFO + S-LA group and the T-AOC, GSH-Px, and CAT activities in the OFO + R-LA group were significantly increased (*p* < 0.05). Meanwhile, the MDA level in the OFO + R-LA group was lower than that in the OFO + S-LA group (*p* < 0.01).

### 3.6. Effect of S-LA and R-LA on Oviduct Structure of Laying Hens under Oxidative Stress

**Figure 6:**
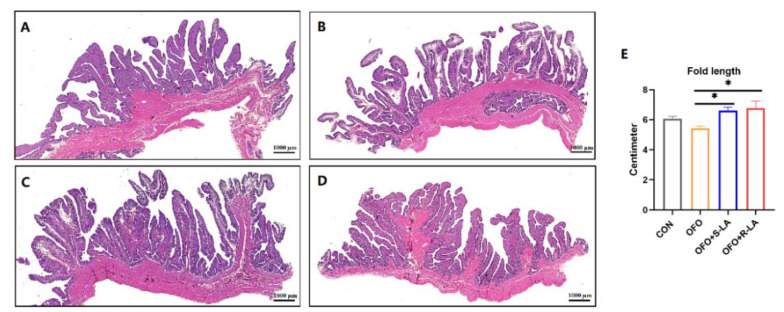
Effect of different treatments on oviduct structure of laying hens. (**A**) CON group; (**B**) OFO group; (**C**) OFO + S-LA group; (**D**) OFO + R-LA group; (**E**) fold length in different treatments. * Indicates minimum significant differences (*p* < 0.05). Graphs were observed at 0.5×, the size unit of the photograph is 1000 µm.

As shown in Figure 6A, the tubular glands in the CON group are tightly packed into the mucosal folds. In the OFO group, the ciliated cells are sparsely distributed, and the tubular glands are sparsely packed into the mucosal folds (Figure 6B). Compared with the OFO group, the ciliated cells are tightly arranged in the S-LA and R-LA group, the fold lengths are significantly increased, the tubular glands are tightly packed into the mucosal folds, and the glands are lined with cells filled with fine eosinophilic granules (Figure 6C–E). In addition, the ciliated cells and tubular gland cells in the R-LA group are more neatly rehearsed and tightly packed than those in the S-LA group (Figure 6C,D). However, comparing the fold lengths in Figure 6E, there was no significant difference between R-LA and S-LA.

### 3.7. Effect of S-LA and R-LA on Oviduct Antioxidant of Laying Hens under Oxidative Stress

**Figure 7:**
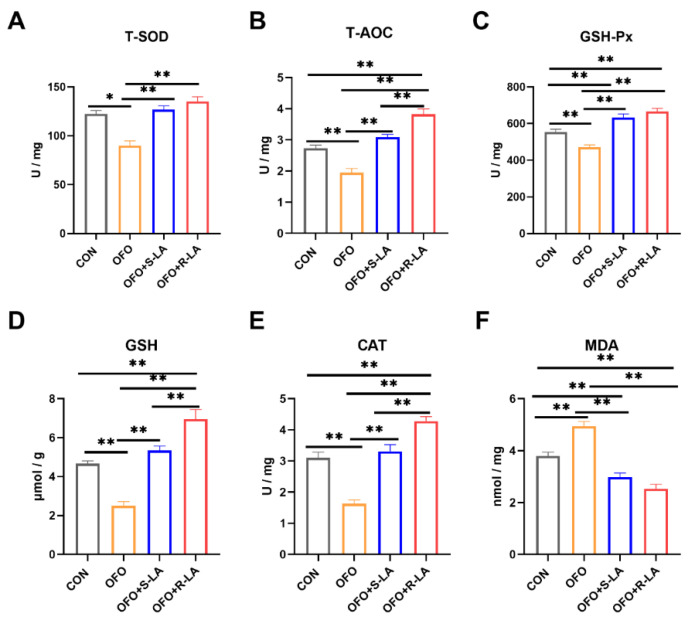
Effect of different treatments on oviduct antioxidant of laying hens. (**A**) Total superoxide dismutase (T-SOD); (**B**) total antioxidant capacity (T-AOC); (**C**) glutathione peroxidase (GSH-Px); (**D**) glutathione (GSH); (**E**) catalase (CAT); (**F**) malondialdehyde (MDA). * Indicates minimum significant differences (*p* < 0.05), and ** indicates highly significant differences (*p* < 0.01).

The addition of the oxidized fish oil significantly increased the activities of the oviduct T-SOD, GSH, and CAT and decreased the MDA levels compared to the addition of fresh fish oil in Figure 7. Compared with the OFO group, the serum T-AOC, T-SOD, GSH-Px, GSH, and CAT activities were all significantly increased, and the serum MDA level was significantly decreased in the OFO + S-LA group and the OFO + R-LA group (*p* < 0.01). Moreover, compared with the CON group, the activities of the GSH-Px and GSH in the OFO + S-LA group and the activities of the T-AOC, GSH-Px, and GSH in the OFO + R-LA group were significantly increased (*p* < 0.01). However, the CAT activity in the OFO + S-LA group was significantly decreased compared to the CON group (*p* < 0.01). In addition, the MDA level in the OFO + R-LA group was lower than that in the CON group (*p* < 0.01). The activities of the T-AOC and CAT in the OFO + R-LA group were higher than that in the OFO + S-LA group (*p* < 0.01).

## 4. Discussion

The two enantiomers of chiral drugs have the same chemical structure, but they generally exhibit differences in pharmacology, toxicology, pharmacokinetics, and metabolism [23,24]. Therefore, the selection of the correct enantiomer is important for health and production. Fendiline is an effective anti-anginal drug with a stronger vasodilatory effect of its R-enantiomer than its S-enantiomer in the treatment of coronary heart diseases [24,25]. Researchers suggested that R-verapamil is best used as a modulator of multidrug resistance in cancer chemotherapy, while S-verapamil is best used as a calcium channel blocker for cardiovascular therapy due to its R-enantiomer being far less cardiotoxic than the S-enantiomer [23,26]. It has been demonstrated above that a different enantiomer of the same chiral drug may differ significantly in terms of biological activity. ALA has one chiral center, so it also exists as two enantiomers: the R-form and S-form [27]. For ALA, it is generally accepted that the R-enantiomer is more biologically active than the S-enantiomer because the naturally occurring ALA is the R-form [28]. Pyruvate decarboxylation is an important step in the metabolic pathway of the glycolysis and tricarboxylic acid cycle. In the pyruvate decarboxylation reaction of intact cells, it is not inhibited by R-LA, while it is inhibited by S-LA [29]. In addition, R-LA may exert more rapid and well-defined biological activity than S-LA, leading to metabolic differences between R- and S-LA [27,30]. In animal models of stroke and in cell culture systems, R-LA inhibited the hydrogen peroxide-induced decrease in cell viability and reduced the concentration of intracellular reactive oxygen species, while S-LA alleviated the effect significantly less than R-LA [31,32]. Similar to our trial, R-LA was significantly more effective than S-LA in alleviating the inflammation response and oxidant stress in laying hens. These results fill some gaps regarding the anti-inflammatory and antioxidant properties of the different enantiomers of ALA on laying hens. The above findings suggest that R-LA may be more effective than S-LA in terms of bioactivity, anti-inflammatory and antioxidant effects, and even in other aspects.

Oxidative stress can be responsible for several pathological diseases that affect different tissues and organs and is one of the underlying factors that can be harmful to overall health [33,34]. Oxidative stress can reduce broilers’ production performance by damaging the liver, intestine, and other tissues and organs [35]. It is reported that oxidative stress caused by environmental toxins and other stressors could impair the health and production performance of laying hens [36]. In animal experimental studies, oxidative stress models are generally constructed by adding oxidized oils, including oxidized fish oil, oxidized corn oil, and oxidized soybean oil. In a test of the effect of using different ratios of oxidized corn oil on broiler chickens, Zhang et al. found that serum and jejunal antioxidant factors showed a significant quadratic response with the increase in oxidized oil concentration [37]. Another trial using oxidized tuna oil as a stressor found that dietary oxidized tuna oil increased some lipid peroxidation markers in trout [38]. In this study, we used oxidized fish oil to induce an oxidative stress model in laying hens. Zhou et al. have found that diets supplemented with 20 g/kg of oxidized fish oil significantly decreased the production performance of birds from 7 to 12 weeks of the experiment and reduced the ileal mucosal secretory immunoglobulin A (sIgA) content. However, there was no effect on the productive performance from 1 to 6 weeks of the experiment [39]. Song et al. found that the addition of 2% oxidized fish oil had no effect on the final body weight (FBW) and weight gain ratio (WGR) in Megalobrama amblycephala, but the addition of 4% oxidized fish oil had a significant negative effect on the production performance [40]. From our results, 1% oxidized fish oil could lead to an increase in serum inflammatory factors and a decrease in oviductal antioxidant factors but did not negatively affect production performance. This may be due to the low amount of oxidized fish oil added or the short duration of the addition, which only leads to inflammation and oxidative damage to the body and tissues without causing substantial harm to production.

In laying hens’ breeding and production, multiple antioxidants such as polyphenols, flavonoids, and vitamins have been used to alleviate oxidative stress in order to maintain the health of laying hens in production [41,42,43]. Likewise, ALA is a multifunctional antioxidant that can scavenge free radicals [44,45]. They are widely used in production because they have an amphiphilic property and can also interact with other antioxidants without any serious side effects when used [46]. Dai et al. indicated that dietary supplementation with 600 mg/kg ALA alone could improve the activity of the SOD and T-AOC and decrease the MDA levels in hens’ ovarian tissue [45]. It is generally believed that ALA is mostly used to alleviate tissue and organ damage caused by oxidative stress and to mitigate the negative effects on the health of the organism. In the present study, our data showed that the dietary addition of ALA alleviated OFO-induced reductions in serum and oviductal antioxidant factors. In a similar vein, Li et al. found that ALA alleviated deltamethrin-induced oxidative stress of Channa argus by activating the Nrf2 signaling pathway [47]. In a trial with rabbits exposed to oxidized olive oil, the dietary addition of ALA was found to have a positive effect on oxidative system parameters and the liver morphological structure [48]. Moreover, another study found that ALA prevents the oxidative stress development of lipopolysaccharides (LPS) induced ventricularly and atrially by reducing the lipid peroxidation and scavenging free radicals [49]. In summary, our data demonstrated that ALA, either S-LA or R-LA, can alleviate the oxidative damage of the organism caused by exogenous oxidative stress.

Moreover, our study found that ALA mitigated the increase in serum inflammatory factors (TNF-α, IL-1β, IL-6, and IFN-γ) induced by oxidized fish oil. Inflammation is considered to interact with oxidative stress. Inflammation increases oxidative damage, which in turn aggravates inflammation [50,51]. The use of antioxidants can not only alleviate oxidative damage but also alleviate the inflammatory response. Considerable evidence suggests that ALA can regulate the mechanism of inflammation [52,53]. In the model of mouse inflammatory bowel diseases (IBD), ALA was found to reduce the plasma levels of LPS and systemic inflammation in mice with colitis [54]. In human health-related studies, ALA was found to reduce the TNF-α and IL-6 levels in many organs and tissues through its anti-inflammatory properties [49,55]. Another study indicated a beneficial effect of ALA administration by reducing the serum and liver levels of TNF-α and IL-1β in rabbits fed oxidized nutrition oils [48]. In addition, ALA reduced the oxidative stress and inflammatory response of the ovary by alleviating the loss of mitochondrial function [56]. Numerous studies have demonstrated that ALA may protect against stress-induced organism and tissue damage via antioxidant and anti-inflammatory properties [15,57]. However, there are relatively few studies on the anti-inflammatory aspects of different enantiomers of ALA in laying hens, and our experimental results fill the gap for the use of ALA in laying hen breeding.

E2 and P are the important hormones of the reproductive system and are essential for the maturation of many fetal organs and the maintenance of the maternal reproductive system [58,59]. Our experiment found that treatment with ALA improved the changes in the serum hormones induced by OFO. These findings correlated with Othman, who demonstrated that ALA exerts antioxidant effects to maintain normal hypothalamic–testicular axis function, which leads to normal androgen secretion [60,61]. Our experimental results might be attributed to an antioxidant milieu of ALA which maintained the normal function of the hypothalamic–oviduct axis, leading to normal estrogen secretion. The above results showed that different enantiomers of ALA exert antioxidant and anti-inflammatory capacities and can also be used to improve the production and reproductive health in laying hens.

## 5. Conclusions

Overall, our present investigation revealed that the addition of oxidized fish oil to the diet could lead to an inflammation response and oxidative damage in aged laying hens, while the addition of ALA could mitigate the negative effects on the serum and oviduct caused by oxidized fish oil. In addition, R-LA was better than S-LA in alleviating inflammation and oxidative damage in laying hens. Thus, these findings will contribute to further understanding the role of different enantiomers of ALA on laying hens’ health and reproduction, which are beneficial for establishing a novel additive for laying hens’ production.

## Figures and Tables

**Table 1:** Effect of different treatments on breeding performance of laying hens.

	Egg Laying Rate/%	Egg Weight/g	Egg Production/g/d	Feed Intake/g	Weight of Laying Hen/kg	Feed/Egg
CON	73.51 ^a,b^	64.80	46.16	114.21	1.63	2.49
OFO	61.21 ^a^	66.42	46.95	115.88	1.66	2.52
OFO + S-LA	67.86 ^a,b^	66.71	47.59	117.32	1.75	2.62
OFO + R-LA	77.53 ^b^	69.70	47.98	118.60	1.82	2.71
*p* value	0.057	0.229	0.956	0.414	0.096	0.81
SEM	2.29	0.85	1.17	0.95	0.029	0.085

^a,b^ Means with different superscripts within a column differ significantly (*p* < 0.05).

## Data Availability

The data are contained within the article and Supplementary Materials.

## Supplementary Materials

The following supporting information can be downloaded at: https://www.mdpi.com/article/10.3390/antiox11081530/s1, Table S1. Composition and nutrient levels of the basal diet (air-dry basis).
