# Supplementary material for: R- Is Superior to S-Form of α-Lipoic Acid in Anti-Inflammatory and Antioxidant Effects in Laying Hens
Source: Antioxidants (Basel). 2022 Aug 5;11(8):1530. doi: 10.3390/antiox11081530 (PMC9405457; doi:10.3390/antiox11081530)
Supplement: Supplementary file 1 [file antioxidants-11-01530-s001.zip › antioxidants-1838364-supplementary.pdf]

Table S1. Composition and nutrient levels of the basal diet (air-dry basis).

Ingredients (%)	Composition	Nutrient levels (%)	Value
Corn	64.60	Metabolic energy (Kcal/kg)	2690
Soybean meal	24.20	Crude Protein	16.04
calcium hydrophosphate	1.70	Calcicum	3.60
Limestone	8.20	Available phosphorus (%)	0.39
Sodium chloride	0.30	Methionine	0.38
DL-methionine (98%)	0.12	Sulfur-containing amino acid	0.65
Choline chloride (50%)	0.10	Lysine	0.78
Vitamin premix[a]	0.04	Threonine	0.59
Trace elements premix[b]	0.30		
Ethoxyquin (60%)	0.02		
Zeolite powder + Lipoic acid	0.42		

[a]premix provided the following per kilogram of the diet: vitamin A, 11700 IU; vitamin D3, 3600IU; vitamin E, 21 IU; vitamin K_3, 4.2 mg; vitamin B_1, 3.0 mg; vitamin B_2, 10.2 mg; folic acid, 0.9 mg; pantothenic acid calcium, 15.0 mg; niacin, 45.0 mg; vitamin B_6, 5.4 mg; vitamin B_{12}, 24 μg; biotin, 0.15 mg.

[b]premix provided the following per kilogram of the diet: Cu (as copper sulfate) 6.00 mg; Fe (as ferrous sulfate) 60.0 mg; Mn (as manganese sulfate) 80.0 mg; Zn (as zinc sulfate) 83.0 mg; Se (as sodium selenite) 0.30 mg; I (as potassium iodide) 1.00 mg.
